# Inverse association between atherogenic index of plasma and testosterone in US adult males: A large cross-sectional study from the NAHNES 2011–2016

**DOI:** 10.3389/fphys.2025.1504778

**Published:** 2025-02-21

**Authors:** Tanjian Li, Lulu Chen, Xin Liang, Xinya Li, Yaqin Li, Yuting Huang, Yu Wang

**Affiliations:** ^1^ School of Nursing, Jinan University, Guangzhou, Guangdong, China; ^2^ Department of Stomatology, Shenzhen Children’s Hosiptal, Shenzhen, Gaundong, China; ^3^ Jinan Community Health Service Center, The First Affiliated Hospital of Jinan University, Guangzhou, Guangdong, China

**Keywords:** NHANES, atherogenic index of plasma, testosterone, testosterone deficiency, male

## Abstract

**Background and objectives:**

The atherogenic index of plasma (AIP), defined as log10 (triglycerides/high-density lipoprotein cholesterol), serves as a biomarker for atherosclerosis and cardiovascular disease (CVD). It is also associated with conditions such as type 2 diabetes, insulin resistance, depression, and both cardiovascular and overall mortality. Serum lipids have been proven to influence serum testosterone levels, and AIP is a significant marker of lipid levels. We hypothesize that AIP may have a specific relationship with testosterone. This article aims to evaluate the correlation between AIP and total testosterone (TT), as well as testosterone deficiency (TD), among the U.S. population.

**Methods:**

Data were collected from the National Health and Nutrition Examination Survey (NHANES) database between 2011 and 2016. This study was categorized into four groups based on the quartiles of AIP. Weighted multivariate linear regression and logistic regression were utilized to evaluate the relationships between AIP and TT, TD. Restricted cubic spline (RCS) was used to investigate the non-linear association between AIP and TT and TD. The subgroup analysis method was employed to investigate the relationships between AIP and TT, TD across various stratifications. Ultimately, the sensitivity study involved a comparison of weighted and unweighted data analyses to ascertain the stability of the conclusions.

**Results:**

A total of 2,572 participants were included in the final study. After adjusting for all confounding factors, multivariate linear regression showed that AIP was independently negatively associated with TT (β = −93.42, 95%CI: −123.66, −63.18, P < 0.001), and multivariate logistic regression showed that AIP level was associated with higher risk of TD (OR = 3.45, 95%CI: 2.09, 5.69, P < 0.001). In the quartile of AIP, TT levels decreased the most (β = −74.81, 95%CI: −105.27, −44.35, p < 0.001) and the risk of TD was highest (OR = 2.89, 95%CI: 1.70, 4.93, p < 0.001). In addition, stratified analyses showed similar results in all subgroups except those with diabetes (P for interaction >0.05 for all comparisons). The final sensitivity analysis revealed that elevated AIP were also associated with decreased TT (β = −101.74, 95%CI: −123.18, −80.3, P < 0.001) and increased incidence of TD (OR = 3.01, 95%CI: 2.17, 4.17, P < 0.001) on unweighted data.

**Conclusion:**

Increased levels of AIP correlate with decreased TT levels and a higher prevalence of TD. Additional research is necessary to investigate the underlying mechanisms connecting them.

## 1 Introduction

Testosterone, a steroid hormone synthesized mainly in Leydig cells of the testis, is essential for various physiological processes, including the development of secondary sexual characteristics in males, the maintenance of muscle strength and growth, sexual function, metabolic and cardiovascular health, and bone mineral density (Iliescu and Reckelhoff, 2006; Dimopoulou et al., 2018; Mohamad et al., 2016; Griggs et al., 1989; Killinger, 1970). Low serum testosterone levels in men can result in dysfunction across multiple organ systems. Low testosterone is associated with decreased libido, erectile dysfunction, and muscle weakness (Rastrelli et al., 2018; O'Connell and Wu, 2014). Furthermore, it can contribute to or worsen metabolic diseases, including metabolic syndrome and osteoporosis (Kupelian et al., 2006; Walsh and Eastell, 2013). Men exhibiting symptoms of testosterone deficiency are at an increased risk for developing coronary artery disease, type 2 diabetes, and hypertension (Colangelo et al., 2009; Ruige et al., 2011). Testosterone deficiency is prevalent, impacting approximately 30% of men between the ages of 40 and 79. Its occurrence escalates with advancing age and is associated with various medical conditions, including obesity, diabetes, and hypertension (Traish et al., 2011). This is projected to rise in the coming decades due to increasing life expectancy (Halpern and Brannigan, 2019). Testosterone deficiency is increasingly recognized as a global issue.

AIP was introduced by Dobiasova and Frohlich in 2001 as a biomarker for plasma atherosclerosis (Dobiásová and Frohlich, 2001). It integrates triglycerides (TG) and high-density lipoprotein cholesterol (HDL-C) levels, reflecting both the particle size of lipoproteins and the log (TG/HDL-C). This ratio serves as a more precise indicator of the specificity and pathogenicity of dyslipidemia (Fernández-Macías et al., 2019). Numerous studies indicate that elevated AIP is significantly linked to cardiovascular disease (CVD) mortality, overall mortality, and hypertensive populations (Qin and Chen, 2024; Duiyimuhan and Maimaiti, 2023). Additionally, AIP serves as a reliable predictor of cardiovascular events and mortality resulting from these events (Onat et al., 2010; Dobiásová, 2006).

Numerous clinical studies have demonstrated a biological link between lipids and sex hormones, revealing an association between TG and HDL-C with TT: Heller identified a consistently positive correlation between HDL-C and TT concentration in a sample of 295 middle-aged men (Heller et al., 1983); Sook et al. reported that among 8,606 Korean male workers, those with low testosterone levels exhibited a higher likelihood of hypertriglyceridemia (Sung et al., 2019); a meta-analysis confirmed a significant association between low testosterone levels and hypertriglyceridemia (Brand et al., 2014); Chung et al. examined 1,055 Korean men aged 45 years and older, revealing a negative correlation between the TG/HDL ratio and TT levels (Chung et al., 2020).

Nonetheless, the existing research presents certain limitations. The current studies are restricted to specific elderly or regional populations, leaving the relationship with the overall male population ambiguous. No studies effectively combine TG and HDL-C to examine their relationship with testosterone. Despite extensive research on the relationship between AIP and CVD, there is a lack of data specifically examining the association between AIP and testosterone levels. The relationship between lipid metabolism and sex hormones suggests a correlation between AIP and testosterone.

This study addresses knowledge gaps by utilizing a comprehensive NHANES dataset and conducting a cross-sectional survey to evaluate the relationship between AIP and TT and TD in adult men in the US. We postulated that there would be a negative and positive correlation, respectively, between AIP and TT and the occurrence of TD.

## 2 Methods

### 2.1 Database and survey populations

**FIGURE 1:**
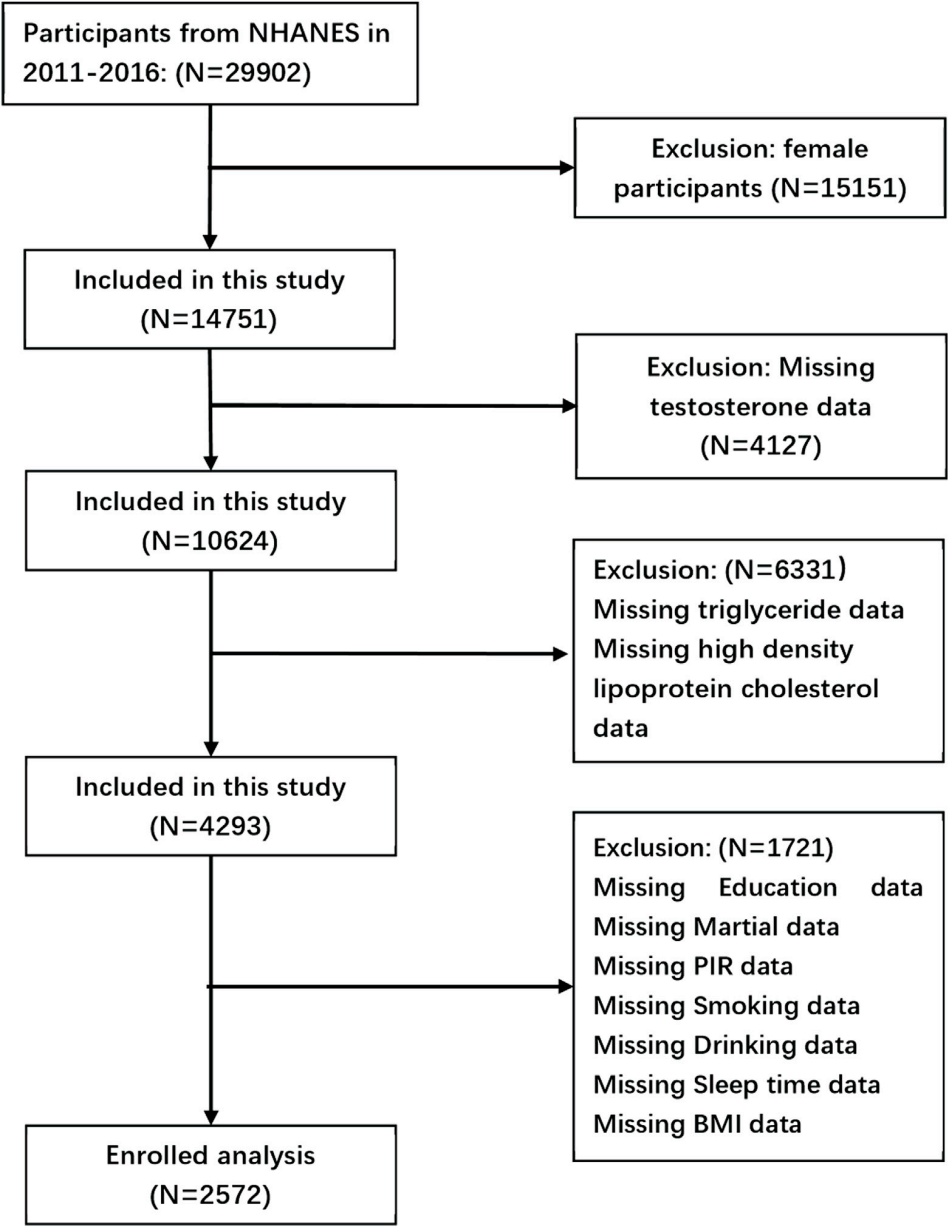
Flow chart of the screening process for the selection of eligible participants.

Data were utilized from the 2011–2016 cycles of the NHANES. The National Center for Health Statistics of the Centers for Disease Control and Prevention conducts the NHANES, which employs a multistage, complex summary, stratified probability sampling design to select representative samples of adults and children from the noninstitutionalized U.S. population for the assessment of their nutritional status. 29,902 participants were included in these cycles. The selected cycles are based on the availability of data regarding total testosterone levels, which is limited to the period from 2011 to 2016. The analysis excluded 15,151 female subjects, 4,127 subjects with incomplete total testosterone data, 6,331 subjects lacking data necessary for AIP calculation, and 1,721 subjects with missing covariate data. Ultimately, 2,572 subjects were included in the final analysis (Figure 1).

### 2.2 Informed consent

NHANES is a dataset that is accessible to the public. Before starting any official inquiry, all participants must give both written and verbal agreement to take part in the research. The study has undergone evaluation and received approval from the ethical review committee of the National Centre for Health Statistics (NCHS). The NCHS IRB/ERB protocol number for 2011–2016 was #2011–17. The website (https://www.cdc.gov/nchs/nhanes/) provides access to all pertinent data.

### 2.3 AIP evaluation

AIP was defined as a LOG10 (TG/HDL-C) ratio (Onat et al., 2010).

### 2.4 Assessment of outcome—TT and TD

According to American Urological Association guidelines, TD are characterized by serum testosterone levels <300 ng/dL (Mulhall et al., 2018). The Centres for Disease Control and Prevention (CDC) developed an isotope dilution liquid chromatography tandem mass spectrometry (ID-LC-MS/MS) technique to measure the levels of total testosterone in serum for routine examination. This method has been particularly designed for samples with a high rate of flow, and has consistently shown a high level of accuracy and precision over a long period of time. The technique has received certification from the CDC Hormone Standardisation Programme (HoSt) and can be traced back to certified reference material acquired from the Australian National Measurement Institute (ANMI) M914 for testosterone. For a comprehensive examination of quality control and quality assurance in the NHANES laboratory and medical technical personnel manual of procedure (LPM), please refer to the following link: https://wwwn.cdc.gov/nchs/data/nhanes/2013-2014/labmethods/TST_H_MET_Tota L_Estradiol_and_Total_Testosterone.

### 2.5 Definition of other variables

The multivariate model considered potential variables that may confound the association between AIP and testosterone, as indicated by prior studies (Cao et al., 2024; Guo et al., 2024; He et al., 2024; Hernández-Pérez et al., 2024). The covariates were age, BMI (≤24.9, 25–29.9, ≥30 kg/m^2^), race (non-Hispanic white, non-Hispanic black, Mexican American, other Hispanic, other races), marital status, family income to poverty ratio (PIR), smoking status, drinking status, education status (below high school, completed high school, high school above), hypertension, diabetes, physical activity, and sleep time (<7 h, 7–9 h, >9 h). Smoking status was classified as never, former, or current smoker according to “at least 100 cigarettes in your lifetime” and “are you a current smoker”. Alcohol consumption was determined according to “at least 12 alcoholic beverages per year?”, “At least 12 alcoholic drinks in your lifetime? “And” frequency of alcohol consumption in the past 12 months “to classify as never, former, and current drinking”.

Hypertension is diagnosed based on several factors, including a history of previous diagnosis of the condition, current use of medication to lower blood pressure, or exhibited systolic or diastolic blood pressure ≥140/90 mmHg. Participants were classified as having diabetes if they received a diagnosis from a physician, exhibited a fasting plasma glucose level of ≥126 mg/dL, utilized insulin or medication for glycemic management, or had a glycated hemoglobin level of ≥6.5. Physical activity was classified into vigorous, moderate, and inactive, based on the subjects’ engagement in activities that induced profuse sweating or a significant increase in breathing or heart rate, as well as those that led to slight sweating or a moderate increase in heart rate. We considered vigorous physical activity if participant reported to do any activity that caused heavy sweating or large increases in breathing or heart rate (e.g., swimming, aerobics, or fast cycling). Moderate physical activity included activities that caused light sweating or a moderate increase in the heart rate, such as playing golf, dancing, bicycling for pleasure, or walking.

### 2.6 Statistical analyses

As recommended by the NHANES Guidelines (Chen et al., 2018; Chen et al., 2020), appropriate weighting techniques were used to address the intricacies of the sample design to ensure that the obtained data were representative at the national level. All data analyses in this study were weighted appropriately, following the rigorous methodology outlined in the official NHANES documentation (https://wwwn.cdc.gov/nchs/nhanes/tutorials/weighting.aspx).

In this study, continuous data were represented as weighted means ± standard deviations, whilst categorical variables were represented as weighted proportions. The comprehensive AIP data were segmented into quartiles (Qs): Q1 (<−0.234), Q2 (−0.234, −0.012), Q3 (−0.012, 0.221), Q4 (>0.221), with Q1 designated as the minimum value. Disparities among various AIP groups (quartiles) were assessed utilizing chi-square tests for categorical variables and t-tests for continuous ones. Weighted linear regression and logistic regression studies were conducted to evaluate the relationship between AIP and the continuous values of TT levels and TD. Three models were employed in the study to account for covariates: Model 1 was unadjusted, Model 2 was adjusted for age, race, education, PIR, and marital status, while Model 3 included adjustments for BMI, diabetes, hypertension, physical activity, and sleep time, in addition to the adjustments made in Model 2. Subsequently, limited cubic spline curves derived from regression model 3 were employed to investigate any nonlinear association between AIP and testosterone. Furthermore, stratified analyses were conducted based on distinct age categories (20–39, 40–59, ≥60 years), including the history of chronic diseases (hypertension and diabetes), BMI, and physical activity, with their interactions evaluated by log-likelihood ratio tests. Sensitivity tests utilizing unweighted data were conducted to verify the robustness of the weighted estimates. Statistical analyses were conducted using R (http://www.R-project.org, The R Foundation) and Free Statistics software version 1.3. Statistical differences were established with two-tailed p values < 0.05.

## 3 Results

### 3.1 Demographic and clinical characteristics of study participants

A total of 2,572 individuals participated in the study. Table 1 presents the baseline characteristics of each group. The weighted sample of 85, 70, 426 participants across the three survey cycles accurately represents the uninstitutionalized U.S. population, predominantly consisting of non-Hispanic whites (69.81%). The average age of the respondents was 46.66 ± 16.31 years, and the average TT level was 455.06 ± 187.74 ng/dL. Participants in Q4 exhibited the lowest testosterone level of 387.19 ± 158.05 ng/dL. They had a higher propensity for obesity (51.66%) and physical inactivity (52.43%). Statistically significant variations were seen in TT, TG, HDL-C levels, BMI, race, smoking status, diabetes, hypertension, and physical activity among the four groups of AIP patients (P < 0.05). As the AIP level rises, the prevalence of patients with diabetes and hypertension escalates.

**TABLE 1:** Weighted demographic and clinical characteristics in accordance with the AIP level.

Variables	Overall	Q1 (<-0.234)	Q2 (−0.234 to −0.012)	Q3 (−0.012–0.221)	Q4 (>0.221)	*P-value*
	(N = 85,700,426)	(N = 21,238,582)	(N = 21,189,847)	(N = 20,437,414)	(N = 22,834,584)	
Continuous variable, mean ± SD						
Age (years)	46.66 ± 16.31	46.90 ± 18.06	46.19 ± 16.48	46.57 ± 16.53	46.94 ± 14.11	0.8669
TT(ng/dL)	455.06 ± 187.74	521.38 ± 192.99	493.67 ± 183.10	421.93 ± 184.10	387.19 ± 158.05	<0.0001
TG(mmol/L)	1.52 ± 1.27	0.63 ± 0.18	1.01 ± 0.20	1.44 ± 0.29	2.89 ± 1.79	<0.0001
HDL-C(mmol/L)	1.27 ± 0.37	1.64 ± 0.41	1.32 ± 0.22	1.16 ± 0.22	0.97 ± 0.19	<0.0001
Categorical variables, number (%)						
Race (%)						<0.0001
Mexican American	7,180,166 (8.38)	1,224,584 (5.77)	1,776,552 (8.38)	1,850,011 (9.05)	2,329,020 (10.20)	
Non-Hispanic Black	7,878,658.38 (9.19)	3,163,467.04 (14.89)	2,071,196 (9.77)	1,630,421 (7.98)	1,013,575 (4.44)	
Non-Hisoanic White	59,824,599 (69.81)	14,600,367 (68.74)	14,847,330 (70.07)	13,730,640 (67.18)	16,646,261 (72.90)	
Other Hispanic	5,119,679 (5.97)	890,576 (4.19)	1,275,386 (6.02)	1,474,062 (7.21)	1,479,655 (6.48)	
Other Race	5,697,324 (6.65)	1,359,588 (6.40)	1,219,384 (5.75)	1,752,280 (8.57)	1,366,072 (5.98)	
Education (%)						0.6235
Below high school	13,683,737 (15.97)	3,658,157 (17.22)	2,951,211 (13.93)	3,280,540 (16.05)	3,793,829 (16.61)	
Completed high school	19,196,520 (22.40)	5,249,240 (24.72)	4,620,443 (21.80)	4,692,784 (22.96)	4,634,053 (20.29)	
High school above	52,820,168 (61.63)	12,331,185 (58.06)	13,618,192 (64.27)	12,464,090 (60.99)	14,406,702 (63.09)	
PIR (%)						0.1677
≤1.3	17,916,667 (20.91)	4,931,717 (23.22)	3,766,149 (17.77)	4,338,480 (21.23)	4,880,321 (21.37)	
1.31–3.5	30,588,490 (35.69)	6,821,928 (32.12)	7,418,936 (35.01)	8,180,544 (40.03)	8,167,082 (35.77)	
>3.5	37,195,269 (43.40)	9,484,938 (44.66)	10,004,761 (47.21)	7,918,389 (38.74)	9,787,180 (42.86)	
Marital (%)						0.0958
Married/Live with partner	57,999,840 (67.68)	13,612,771 (64.09)	14,049,703 (66.30)	13,984,984 (68.43)	16,352,381 (71.61)	
Widowed/Divorced/Separated	10,979,129 (12.81)	2,365,420 (11.14)	2,966,852 (14.00)	2,611,789 (12.78)	3,035,067 (13.29)	
Never married	16,721,457 (19.51)	5,260,390 (24.77)	4,173,291 (19.69)	3,840,641 (18.79)	3,447,136 (15.10)	
BMI (%)						<0.0001
≤24.9	21,787,335 (25.42)	9,249,412 (43.55)	6,183,460 (29.18)	4,090,252 (20.01)	2,264,211 (9.92)	
25–29.9	33,060,915 (38.58)	7,893,513 (37.17)	9,035,972 (42.64)	7,357,124 (36.00)	8,774,307 (38.43)	
≥30	30,852,176 (36.00)	4,095,657 (19.28)	5,970,415 (28.18)	8,990,038 (43.99)	11,796,066 (51.66)	
Smoke (%)						0.0057
Never	38,477,880 (44.90)	10,514,970 (49.51)	11,008,890 (51.95)	7,491,001 (36.65)	9,463,020 (41.44)	
Former	27,734,492 (32.36)	6,250,331 (29.43)	5,949,443 (28.08)	7,802,279 (38.18)	7,732,440 (33.86)	
Now	19,488,053 (22.74)	4,473,281 (21.06)	4,231,514 (19.97)	5,144,134 (25.17)	5,639,124 (24.70)	
Drink (%)						0.5460
Never	3,124,329 (3.65)	834,643 (3.93)	844,197 (3.98)	549,898 (2.69)	895,591 (3.92)	
Former	9,944,655 (11.60)	1,983,038 (9.34)	2,423,145 (11.44)	2,746,971 (13.44)	2,791,501 (12.22)	
Now	72,631,442 (84.75)	18,420,901 (86.73)	17,922,505 (84.58)	17,140,545 (83.87)	19,147,492 (83.85)	
Diabetes (%)						<0.0001
No	72,704,286 (84.84)	19,337,889 (91.05)	18,739,425 (88.44)	16,732,748 (81.87)	17,894,225 (78.36)	
Yes	12,996,140 (15.16)	1,900,693 (8.95)	2,450,422 (11.56)	3,704,666 (18.13)	4,940,359 (21.64)	
Hypertension (%)						0.0084
No	56,961,370 (66.47)	15,191,276 (71.53)	1,4,531,840 (68.58)	13,836,394 (67.70)	1,3,401,859 (58.69)	
Yes	28,739,057 (33.53)	6,047,306 (28.47)	6,658,007 (31.42)	6,601,020 (32.30)	9,432,724 (41.31)	
Sleep time (%)						0.063
<7 h	25,868,785 (30.19)	5,806,798 (27.34)	7,089,794 (33.46)	6,148,030 (30.08)	6,824,163 (29.89)	
7–9 h	56,489,947 (65.92)	14,192,769 (66.83)	13,388,321 (63.18)	13,339,714 (65.27)	15,569,143 (68.18)	
>9 h	3,341,694 (3.90)	1,239,016 (5.83)	711,731 (3.36)	949,670 (4.65)	441,277 (1.93)	
Physical activity (%)						<0.0001
Inactive	38,010,009 (44.35)	8,390,171 (39.50)	8,708,233 (41.10)	8,939,705 (43.74)	11,971,900 (52.43)	
Moderate	22,213,435 (25.92)	4,444,832 (20.93)	5,441,785 (25.68)	6,301,545 (30.83)	6,025,273 (26.39)	
Vigorous	25,476,982 (29.73)	8,403,579 (39.57)	7,039,829 (33.22)	5,196,163 (25.42)	4,837,412 (21.18)	

Values are presented as mean ± standard deviation or number (%). Abbreviations: AIP, atherogenic index of plasma; Q, quartile; PIR, poverty to family income of ratio; BMI, body mass index; HDL-C, high-density lipoprotein-cholesterol; TG, triglyceride; TT, total testosterone.

### 3.2 The association between AIP and TT, TD

Table 2 shows the 95% confidence intervals of β and OR for the association between AIP and TT and TD in the three regression models.

**TABLE 2:** Association between AIP and TT and TD, weighted.

Exposure	Model1	Model2	Model3
Total testosterone, β (95% CI), *P*-value			
AIP, Continuous	−159.97 (−188.08, −131.86), <0.001	−156.54 (−183.83, −129.25), <0.001	−93.42 (−123.66, −63.18), <0.001
AIP, Quartile			
Q1	Ref	Ref	Ref
Q2	−27.72 (-57.57, 2.14), 0.068	−25.68 (−56.58, 5.22), 0.1	−6.05 (−33.88, 21.77), 0.656
Q3	−99.46 (−125.71, −73.20), <0.001	−97.87 (−123.51, −72.22), <0.001	−58.10 (−84.70, −31.50), <0.001
Q4	−134.19 (-162.69, −105.70), <0.001	−130.62 (−159.03, −102.22), <0.001	−74.81 (−105.27, −44.35), <0.001
*P* for trend	<0.001	<0.001	<0.001
Testosterone deficiency, OR (95% CI); *P*-value			
AIP, Continuous	5.35 (3.68, 7.77), <0.001	5.62 (3.81, 8.28), <0.001	3.45 (2.09, 5.69), <0.001
AIP, Quartile			
Q1	Ref	Ref	Ref
Q2	1.77 (1.10, 2.86), 0.02	1.84 (1.11, 3.04), 0.019	1.54 (0.91, 2.63), 0.104
Q3	2.87 (1.81, 4.56), <0.001	3.00 (1.84, 4.87), <0.001	1.97 (1.09, 3.55), 0.026
Q4	4.49 (3.00, 6.72), <0.001	4.69 (3.06, 7.21), <0.001	2.89 (1.70, 4.93), <0.001
*P* for trend	<0.001	<0.001	<0.001

Abbreviations: Ref, Reference; 95%CI, 95% confidence interval; OR, odds ratio; Ref, reference group; AIP, atherogenic index of plasma.

Model 1 adjust for: None.

Model 2 adjust for: Age, Race, Marital, PIR, education.

Model 3 adjust for: Age, Race, Marital, PIR, education; BMI, status, Smoking status, Drinking status, Hypertension, Diabetes, Sleep Time, Physical Activity.

The findings indicated a significant independent negative correlation between AIP and TT across various adjusted models (model 1, β = −159.97, 95% CI: −188.08, −131.86; model 2, β = −156.54, 95% CI: −183.83, −129.25). Similar results were noted in model 3 after comprehensive adjustment for confounding factors (β = −93.42, 95%CI: −123.66, −63.18; All p < 0.05). In models 1, 2, and 3, the β values for groups Q2, Q3, and Q4 were significantly different from those of Q1 (all p < 0.05). The two highest AIP groups (Q3 and Q4) exhibited significantly elevated testosterone levels compared to the lowest AIP group (Q1).

A weighted multivariate logistic regression model was employed to examine the association between AIP and TD. After full adjustment for all covariates, the analysis of continuous variables indicated that the risk of testosterone deficiency increased by 109% (OR = 3.45, 95% CI: 2.09, 5.69) for each unit increase in AIP. The analysis of categorical variables indicated that the risk of testosterone deficiency increased progressively, peaking at Q4 (OR = 2.89, 95%CI: 1.70, 4.93). In the other models lacking full covariate adjustment, the odds ratios for Q3 and Q4 were significantly different from Q1 (all p < 0.05), with all trend p values also below 0.05.

### 3.3 Linear relationship between AIP and TT, TD

**FIGURE 2:**
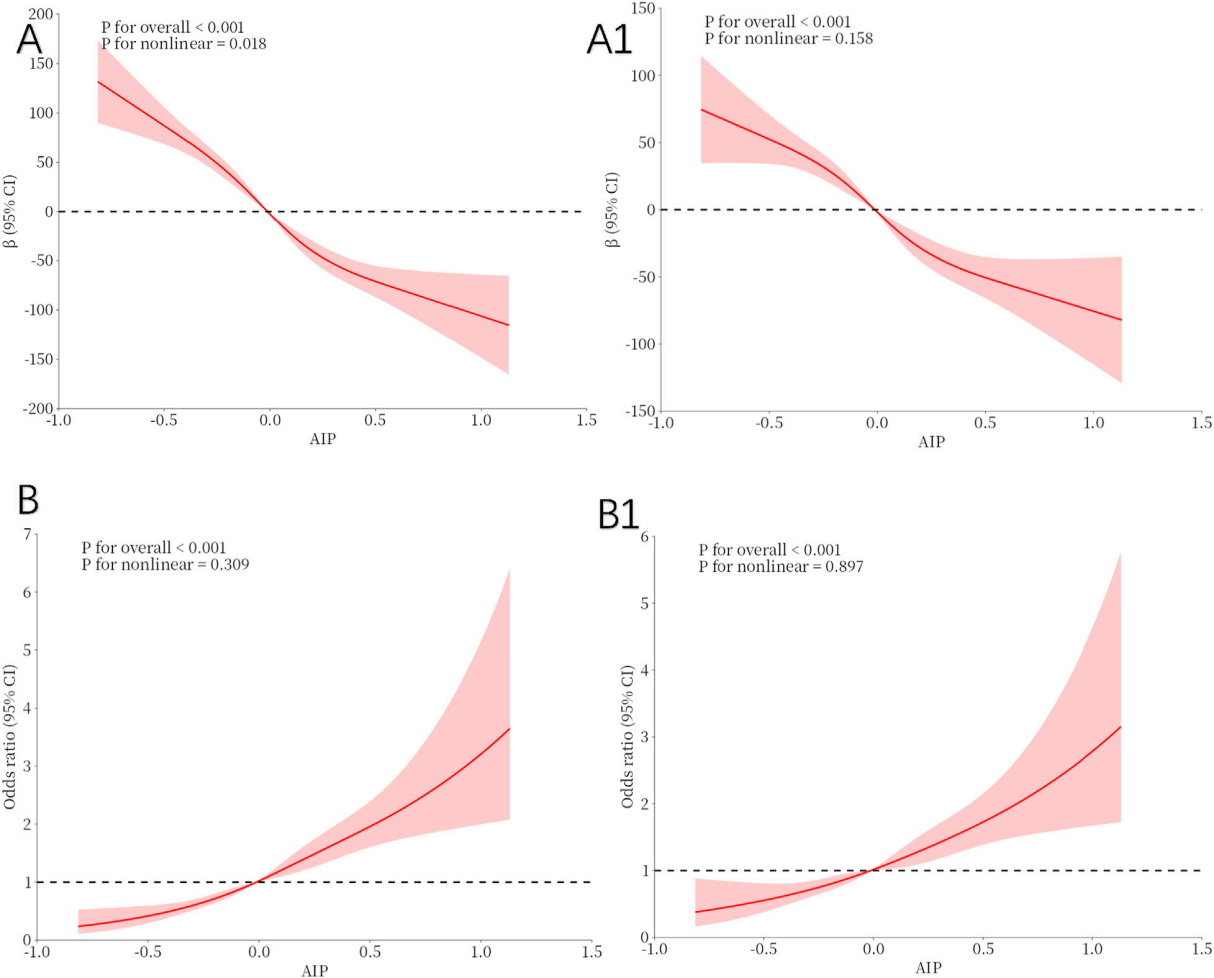
Restricted cubic spline fitting for the association between AIP with testosterone levels. **(A, A1)** illustrate the nonlinear association between the AIP and TT, while **(B, B1)** depict the nonlinear relationship between the AIP and TD. **(A, B)**: No adjustment. **(A1, B1)**: Adjust for Age, Race, Marital, PIR, Education, BMI status, Smoking status, Drinking status, Hypertension, Diabetes, Sleep Time and Physical Activity.

The restricted spline regression model depicted in Figure 2 demonstrated a linear inverse relationship between AIP and TT in the adult male population (p for overall:<0.001), without adjusting for any covariates (Figure 2A). Upon controlling for all confounding factors (Figure 2B), AIP remained inversely associated with TT (p for overall: <0.001). RCS analysis indicated a consistent linear relationship between AIP and TD (Figures 2A1, B1).

### 3.4 Subgroup analysis

Our study investigated the relationship between AIP and dTT and TD across various subgroups. The analysis was adjusted for categorical Age, BMI, Diabetes, Hypertension, and Physical activity.The findings indicated that the correlation between AIP and TT levels, as well as TD, was consistent across all subgroups except for those with diabetes (all p < 0.001). No significant interaction was detected (p > 0.05). The comprehensive findings of the analysis are displayed in Tables 3, 4.

**TABLE 3:** Subgroup analysis for the association between AIP and TT, weighted.

Variables	Beta (95%CI)	*P* Value	P For interaction
Age			0.36
20–39	−279.14 (−394.46, −163.81)	<0.001	
40–59	−202.91 (−281.72, −124.11)	<0.001	
≥60	−226.77 (−388.72, −64.81)	0.01	
BMI			0.253
≤24.9	−247.37 (−369.51, −125.22)	<0.001	
25–29.9	−311.73 (−430.83, −192.62)	<0.001	
≥30	−164.53 (−272.16, −56.91)	0.004	
Diabetes			0.506
No	−254.49 (−334.11, −174.87)	<0.001	
Yes	−180.26 (−384.95, 24.43)	0.08	
Hypertension			0.785
No	−226.50 (−312.76, −140.24)	<0.001	
Yes	−285.26 (−393.57, −176.96)	<0.001	
Physical activity			0.305
Inactive	−214.73 (−299.47, −130.00)	<0.001	
Moderate	−316.61 (−447.54, −185.67)	<0.001	
Vigorous	−209.72 (−335.49, −83.95)	0.002	

All stratified factors include gender, age, race, body mass index (BMI), education level, hypertension, diabetes, physical activity, drinking status, smoking status, and sleep time, except the stratified factor itself.

**TABLE 4:** Subgroup analysis for the association between AIP and TD, weighted.

Variables	Or (95%CI)	P Value	P For interaction
Age			0.518
20–39	4.25 (1.49, 12.10)	0.01	
40–59	2.57 (1.30, 5.08)	0.01	
≥60	3.51 (1.27, 9.72)	0.02	
BMI			0.309
≤24.9	7.56 (1.89, 30.18)	0.01	
25–29.9	5.25 (1.99, 13.86)	0.002	
≥30	2.19 (1.07, 4.48)	0.03	
Diabetes			0.332
No	3.85 (2.05, 7.20)	<0.001	
Yes	2.17 (0.68, 6.98)	0.18	
Hypertension			0.126
No	4.05 (2.40, 6.82)	<0.001	
Yes	2.97 (1.47, 5.99)	0.004	
Physical activity			0.587
Inactive	2.51 (1.31, 4.82)	0.01	
Moderate	3.98 (1.37, 11.54)	0.01	
Vigorous	4.68 (1.64, 13.34)	0.01	

All stratified factors include gender, age, race, body mass index (BMI), education level, hypertension, diabetes, physical activity, drinking status, smoking status, and sleep time, except the stratified factor itself.

### 3.5 Sensitivity analyses

Table 5 illustrates that the correlation between AIP and TT persisted when the analysis was conducted with unweighted data (β = −155.81, p < 0.001) and remained steady in model 2 after adjusting for all covariates (β = −101.74, p < 0.001). Comparable outcomes were noted in the examination of AIP and TD (Model 2: OR = 3.01, p < 0.001).

**TABLE 5:** The comparison between weighted and unweighted analysis for detection of sensitivity.

Model	Weight	Total testosterone (ng/dL)
		β (95%CI), *P*-value
Model 1	Weighted	−159.97 (-188.08, −131.86), <0.001
	Unweighted	−155.81 (−176.64, −134.98), <0.001
Model 2	Weighted	−93.42 (−123.66, −63.18), <0.001
	Unweighted	−101.74 (−123.18, −80.3), <0.001

Model	Weight	Testosterone deficiency
		OR (95% CI), *P-*value
Model 1	Weighted	5.35 (3.68, 7.77), <0.001
	Unweighted	4.23 (3.19, 5.62), <0.001
Model 2	Weighted	3.45 (2.09, 5.69), <0.001
	Unweighted	3.01 (2.17, 4.17), <0.001

Model 1 adjust for: None.

Model 2 adjust for: Age, Race, Marital, PIR, education; BMI, status, Smoking status, Drinking status, Hypertension, Diabetes, Sleep Time and Physical Activity.

## 4 Discussion

This study utilizes a large-scale US population survey to demonstrate, for the first time, an epidemiological relationship between AIP levels and testosterone. This study comprised 2,572 participants, with the sample size representing serum testosterone levels in 857,004 adult men. The study’s results indicate that as AIP increases, total testosterone levels significantly decline, leading to a heightened risk of testosterone deficiency. Sensitivity analysis was conducted to assess the robustness of our findings.

Numerous prior investigations have been conducted regarding lipids and testosterone. Andrade et al. examined the correlation between lipid levels and testicular function in a cohort of 278 infertile men. Their findings indicated that triglycerides (TG) serve as a sensitive marker for male reproductive dysfunction, with elevated serum TG levels correlating with decreased serum total testosterone levels (Andrade et al., 2023). Hamalainen et al. assessed serum sex hormone levels and blood lipids in 30 healthy Finnish men with comparable dietary habits, revealing a positive correlation between serum total testosterone and free testosterone levels and HDL-C (Hämäläinen et al., 1986). Semmens et al. found a significant inverse relationship between testosterone levels and HDL-C in male vegetarian participants, after adjusting for other variables (Semmens et al., 1983). Chung et al. identified an inverse correlation between the TG/HDL ratio and TT in middle-aged and elderly Korean men (Chung et al., 2020). As for AIP, A study involving 280 male patients with type 2 diabetes and 50 control subjects demonstrated a clear inverse relationship between TT levels and the cardiovascular disease risk predictor AIP (Rovira-Llopis et al., 2017).

The findings of our study align with those of prior research. This study utilizes the NHANES database with a large sample population to investigate the association between AIP and testosterone in adult men in the United States. AIP, which integrates both TG and HDL-C, is employed for this analysis. These findings suggest that AIP is linked to testosterone levels in adult males across various races and ages. We hypothesize that AIP may be utilized for the effective management of TT and the prevention of TD. Consequently, further studies on AIP and TT or TD across diverse populations with larger sample sizes are essential to strengthen the evidence base.

The mechanisms contributing to the reduction of AIP and serum total testosterone levels remain unclear; however, several explanations exist. The abnormal AIP index typically indicates dyslipidemia, characterized by elevated triglycerides and reduced HDL-C levels. Abnormal lipid metabolism influences the synthesis and secretion of reproductive hormones. Research involving animal models indicates that a high-fat diet elevates body weight in mice, particularly increasing serum triglyceride levels, which subsequently reduces testosterone levels and contributes to infertility in male mice (Erdemir et al., 2012). A clinical study indicated that alterations in lipid levels influence the secretion of reproductive hormones, specifically testosterone, follicle-stimulating hormone, and luteinizing hormone, with testosterone exhibiting the most significant impact (Yang and Wang, 2016). Bi et al. examined the correlation between lipid levels and serum reproductive hormones in a sample of 885 men, revealing that serum testosterone levels were significantly reduced in hyperlipidemic men compared to those with normal lipid levels (Bi et al., 2024).

AIP, on the other hand, is an indicator utilized to evaluate lipid metabolism and was initially employed to forecast atherosclerosis and CVD risk (Fernández-Macías et al., 2019). Recent studies have demonstrated that AIP serves as a significant biomarker for predicting unfavorable metabolic conditions, including diabetes and insulin resistance (Yin et al., 2023; Zheng et al., 2023), metabolic syndrome (Li et al., 2021), and Visceral Adiposity (Zhou et al., 2018; Shen et al., 2018). A significant quantity of aromatase enzymes present in visceral adipose tissue (VAT) can catalyze the conversion of testosterone to estradiol, leading to increased estradiol levels that activate hypothalamic estrogen receptors, subsequently inhibiting the Hypothalamic-pituitary-gonadal (HPG) axis and influencing testosterone release (Su et al., 2023). Moreover, hyperglycemia can directly induce a reduction in testosterone production by activating the Toll like receptor-4 (TLR-4) mediated oxidative stress pathway (Karpova et al., 2020). The association between testosterone levels and chronic diseases, as well as metabolic disorders, is bidirectional: reduced TT levels result in heightened lipoprotein lipase expression and VAT buildup. The buildup of VAT results in the secretion of proinflammatory cytokines (TNF-α, IL-1β) and leptin, which directly suppresses the HPG axis and Leydig cells in the testes. This leads to reduced serum testosterone synthesis (Pivonello et al., 2019). The accumulation of VAT results in heightened aromatase levels, contributing to insulin resistance and augmenting the risk of type 2 diabetes (Singh et al., 2003). Furthermore, TD has been linked to the development of MetS and to endothelial and mitochondrial dysfunction (Traish and Zitzmann, 2015). Numerous epidemiological studies have established a strong correlation between low serum testosterone levels and detrimental metabolic risk factors, including insulin resistance (Zolla, 2022; Tishova et al., 2024; Minooee et al., 2019), diabetes (Grossmann et al., 2010; Grossmann, 2011), dyslipidemia (Newman, 2023), and metabolic syndrome (Fernández-Miró et al., 2016), through the reciprocal promotion of bidirectional pathophysiological pathways involving various shared biochemical factors, such as proinflammatory pathways and cytokines. Consequently, it may be helpful to explain the mechanism of AIP and testosterone, nevertheless, additional investigations are required to elucidate the further molecular mechanisms of between them.

This study utilized NHANES data to examine the correlation between AIP and testosterone in a large sample of the US population. The chosen index AIP is both straightforward to compute and readily accessible. Furthermore, we included the appropriate sampling weights and design into our statistical analysis to enhance the precision of our representation of the general population. While this constitutes a strength of the study, it also has several limits. Initially, it was a cross-sectional study, indicating that causation couldn't be examined. Due to the numerous factors influencing AIP or testosterone, we may not consider all confounding variables that could impact the relationship between AIP and testosterone levels. Moreover, the absence of sex hormone-binding globulin and free testosterone levels constitutes limitations of our investigation. It is essential to recognize that the NHANES database exclusively represents the US population. The persistence of the relationship between AIP and TT and TD in individuals from various countries warrants investigation.

## 5 Conclusion

This study is, to our knowledge, the inaugural inquiry examining the relationship between AIP and serum testosterone. In the weighted multivariate regression analysis, AIP exhibited a negative correlation with TT and a positive correlation with the risk of TD. Our findings require validation through additional research.

## Data Availability

The original contributions presented in the study are included in the article/Supplementary Material, further inquiries can be directed to the corresponding author.
